# Supplementary material for: Butyrate Lowers Cellular Cholesterol through HDAC Inhibition and Impaired SREBP-2 Signalling
Source: Int J Mol Sci. 2022 Dec 7;23(24):15506. doi: 10.3390/ijms232415506 (PMC9779842; doi:10.3390/ijms232415506)
Supplement: Supplementary file 1 [file ijms-23-15506-s001.zip › ijms-2015569-supplementary.pdf]

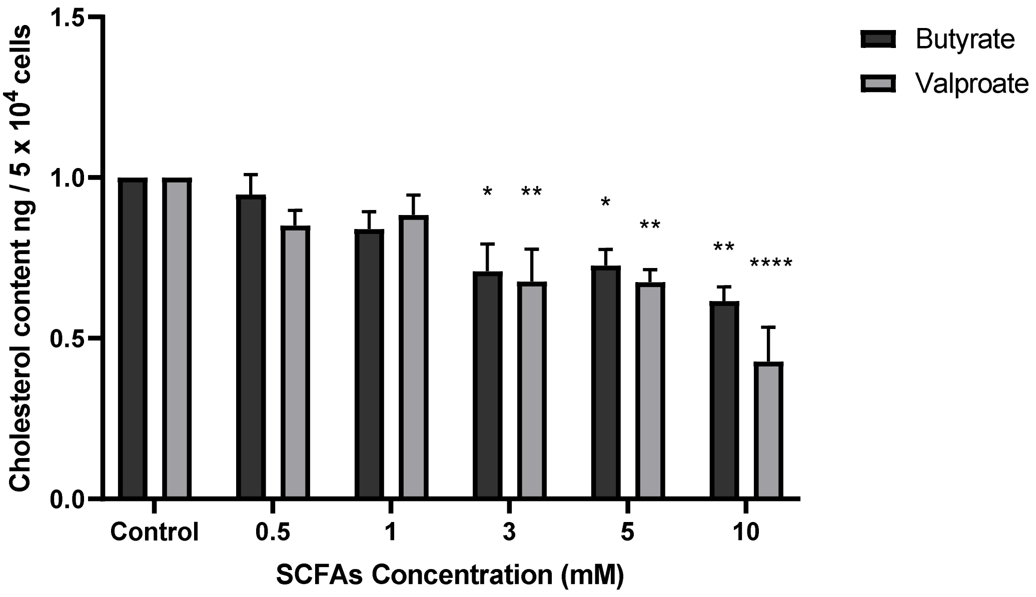

Figure S1. Cellular cholesterol content of HepG2 cells treated with varying concentrations of butyrate or valproate for 24 h. Results represent the mean combined from at least three independent experiments, with error bars representing SEMs. * $p < 0.05$, ** $p < 0.01$, **** $p < 0.0001$.

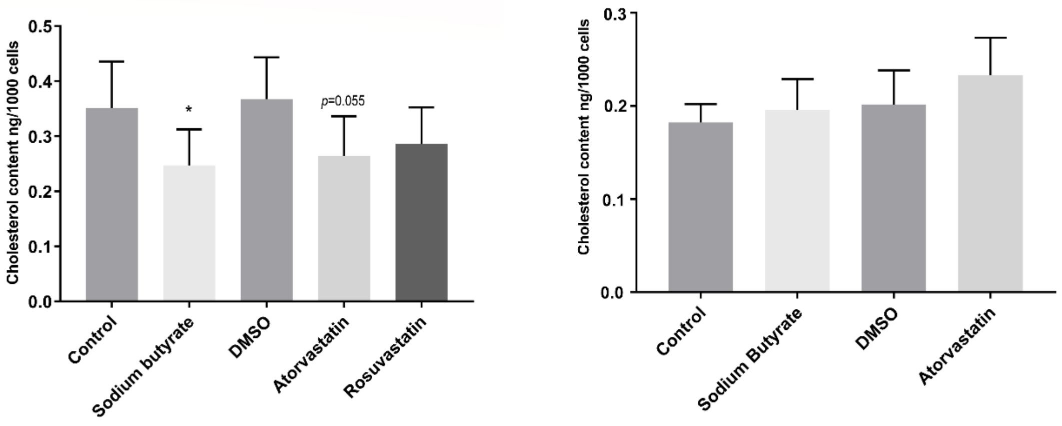

(**a**) (**b**)

Figure S2. (**a**) Cellular cholesterol content of BRIN-BD11 cells; (**b**) Cholesterol content of THP1 cells. Cells were treated with 10 μM statins or 5 mM sodium butyrate for 24 h. * $p < 0.05$.

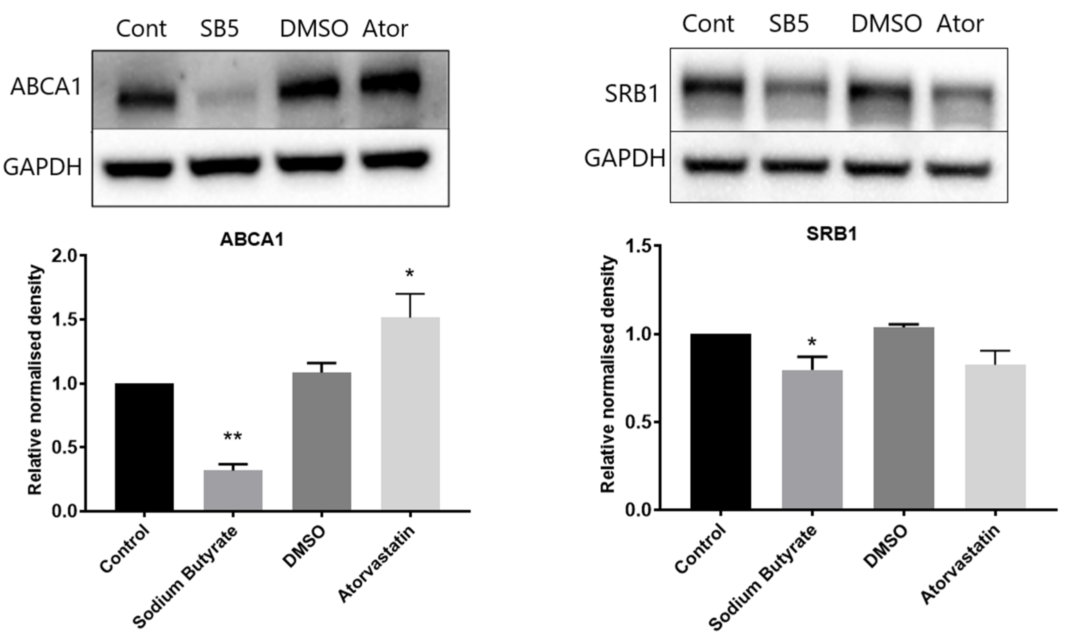

(**a**) (**b**)

Figure S3. (**a**) Immunoblot of ABCA1 in HepG2 cells in HLPS; (**b**) Immunoblot of SRB1 in HepG2 cells in HLPS. Cells were treated with 1 mM (SB1) or 5 mM (SB5) sodium butyrate, or 10 μM atorvastatin for 24 h in media containing HLPS. The graphs represent the mean density readings as normalised to GAPDH from three independent experiments, with error bars representing SEMs. * $p < 0.05$, ** $p < 0.01$ compared to control.

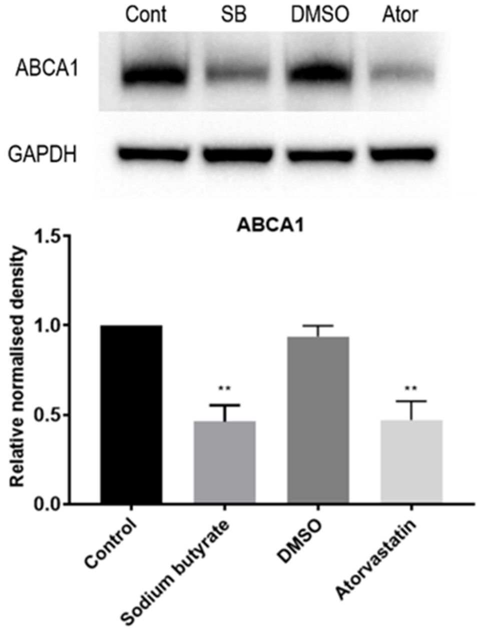

Figure S4. Immunoblot of of ABCA1 in THP-1 cells. Cells were treated with 5 mM sodium butyrate or 10 μM atorvastatin for 24 h. The graph represents the mean combined density readings as normalised to GAPDH from three independent experiments with error bars representing SEMs. ** $p < 0.01$.

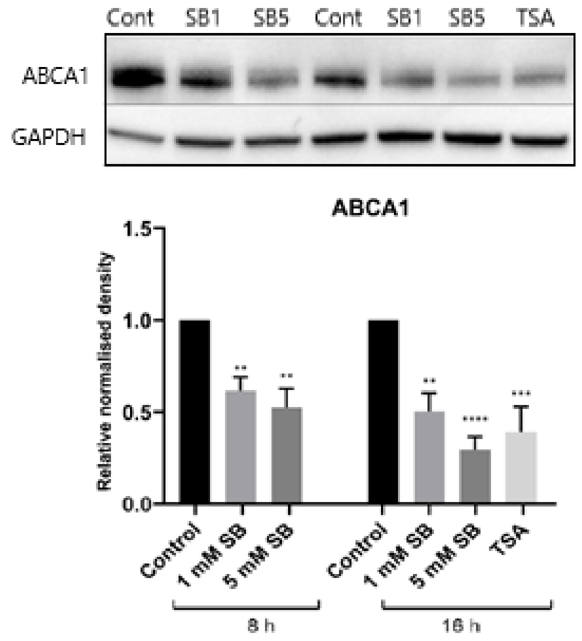

Figure S5. Immunoblot of ABCA1 in HepG2 cells. Cells were treated with 1 mM (SB1) and 5 mM (SB5) sodium butyrate for 8 h and 16 h or TSA for 16 h. The graph represents the mean density readings as normalised to GAPDH from three independent experiments, with error bars representing SEMs. ** $p<0.01$, *** $p < 0.001$, **** $p < 0.0001$.

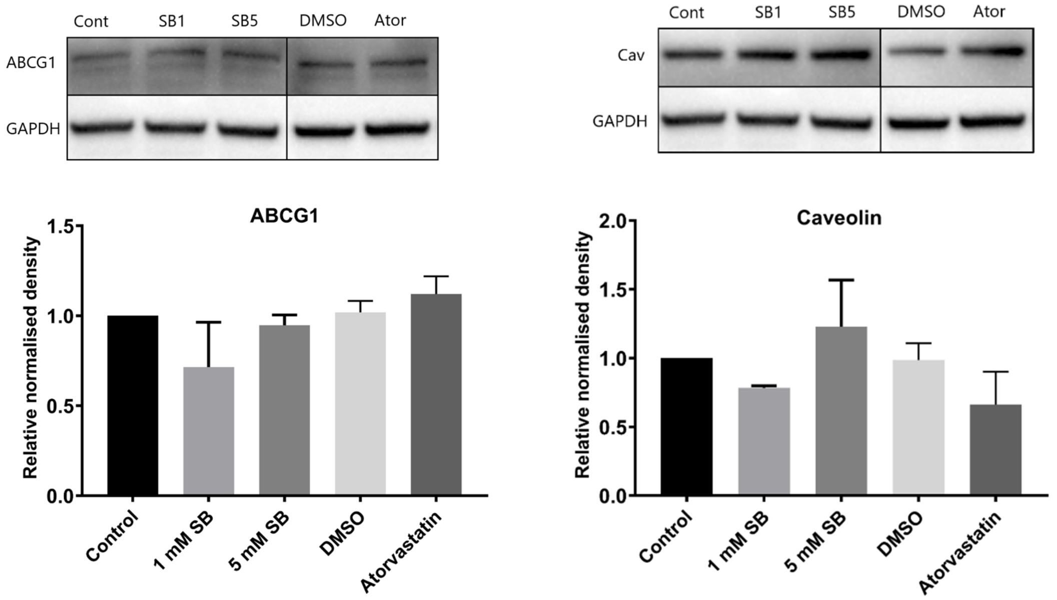

(**a**) (**b**)

Figure S6. (**a**) Immunoblot of ABCG1 in HepG2 cells; (**b**) immunoblot of caveolin in HepG2 cells. Cells were treated with 1 mM (SB1) or 5 mM (SB5) sodium butyrate, or 10 µM atorvastatin for 24 h in media containing HLPS. The graphs represent the mean density readings as normalised to GAPDH from three independent experiments, with error bars representing SEMs.

Table S1. Primers for qPCR experiments

Target	Forward primer	Reverse primer	Annealing temp
GAPDH	ACAGTTGCCATGTAGACC	TTGAGCACGGGTACTTTA	57-60°
YWHAZ	AACTTGACATTGTGGACATC	AAAACTATTTGTGGGACAGC	57-63°
RPL13A	GTCTGAAGCCTACAAGAAAG	TGTCAATTTTCTTCTCCACG	61-63°
LDLR	GAGGACAAAGTATTTTGGACAG	GTAGGTTTTCAGCCAACAAG	57°
PPARG	AAAGAAGCCAACACTAAACC	TGGTCATTTCGTTAAAGGC	63°
SREBF2	CAGCAGGTCAATCATAAACTG	GGACATTCTGATTAAAGTCCTC	61.4°
NR1H3	CATGACCGACTGATGTTC	CAAACACTTGCTCTGAGTG	61.4°
APOA1	AGGAGTACACTAAGAAGCTC	AAACGTTTATTCTGAGCACC	62°
APOB	CTTACATCCTGAACATCAAGAG	AGTTTCCATACACGGTATCC	63°
HMGCR	ACTTCGTGTTCATGACTTTC	GACATAATCATCTTGACCCTC	59°
SREBF1	AATCTGGGTTTTGTGTCTTC	AAAAGTTGTGTACCTTGTGG	61.4°
CAV1	CAGGGACATCTCTACACC	TCAAAGTCAATCTTGACCAC	61.4°
CYP7A1	AAATCTACCCAGACCCTTTG	TTCCAGGACATATTGTAGCTC	58°
ABCG1	GAGGTGAACCCTTTTCTTTG	CCTTTCTCAACCCCTTTAATC	60°
MVK	CATGTTGTCAGAAGTCCTAC	CTCAAGTTCAAGGATACAGC	59°
ABCA1	GTGTTTCTGGATGAACCC	TTCCATTGACCATGATTGC	62°
SCARB1	ACAAAAGCAACATCACCTTC	TGGGCTTATTCTCCATCATC	62°
